# Retrieval technique for a sheared guidewire remnant in the gallbladder duct using a novel basket catheter

**DOI:** 10.1055/a-2432-3302

**Published:** 2024-11-08

**Authors:** Sho Ishikawa, Mitsuhito Koizumi, Masahito Kokubu, Yusuke Okujima, Yuki Numata, Teru Kumagi, Yoichi Hiasa

**Affiliations:** 1 38050 Department of Gastroenterology & Metabology, Ehime University Graduate School of Medicine, Toon, Japan; 2 89456 Post Graduate Medical Education Center, Ehime University Hospital, Toon, Japan

Endoscopic transpapillary gallbladder drainage (ETGBD) is one of the most difficult endoscopic retrograde cholangiopancreatography (ERCP)-related procedures to perform, as it often causes adverse events in the gallbladder duct 1 . Retrieval is technically difficult if a sheared guidewire remnant remains in the gallbladder duct. Herein, we present a case in which a sheared guidewire remnant was successfully retrieved from the gallbladder duct using a novel basket catheter.

A 78-year-old man was admitted to our hospital with acute cholecystitis ( Fig. 1 ). He was promptly treated using antibiotics and ETGBD. While manipulating the guidewire in the gallbladder duct, the guidewire became kinked, broke off, and remained in the duct ( Fig. 2 **a, b** ). We attempted to retrieve the sheared guidewire using a RASEN 2 basket catheter (Kaneka Medical, Osaka, Japan) that was inserted along the guidewire, deployed into the gallbladder duct, and rotated clockwise to confirm that the guidewire remnant was inside ( Fig. 2 **c** ). Once we closed the basket, we were able to firmly grip the guidewire and pull it out of the patient through the channel of the endoscope ( Fig. 2 **d** , Fig. 3 ).

**Fig. 1:**
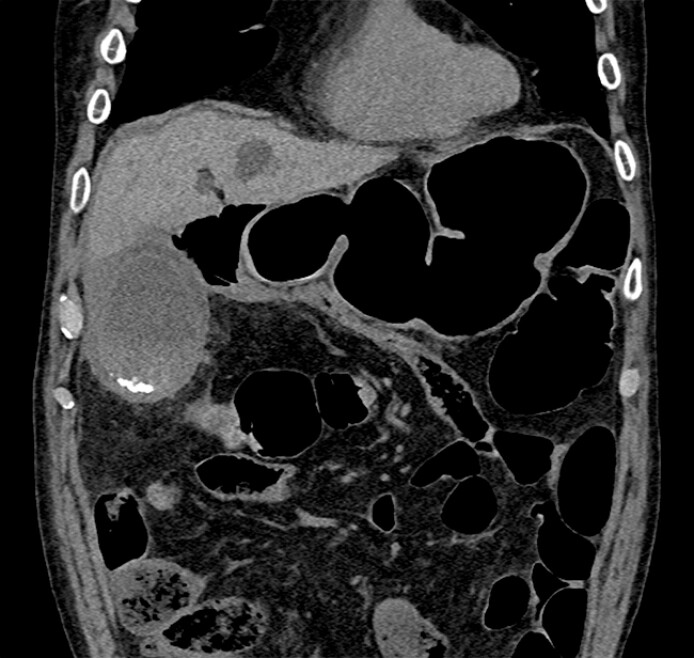
Computed tomography image showing acute cholecystitis on the patient’s day of admission.

**Fig. 2:**
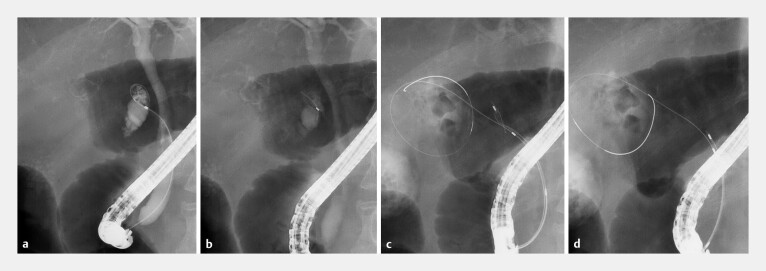
Fluoroscopic images of endoscopic transpapillary gallbladder drainage procedure. **a** Guidewire manipulation in the gallbladder duct. **b** Sheared guidewire remnant in the gallbladder duct. **c** A basket catheter was inserted into the gallbladder duct along the guidewire. **d** Successful grasping and retrieval of the sheared guidewire remnant.

**Fig. 3:**
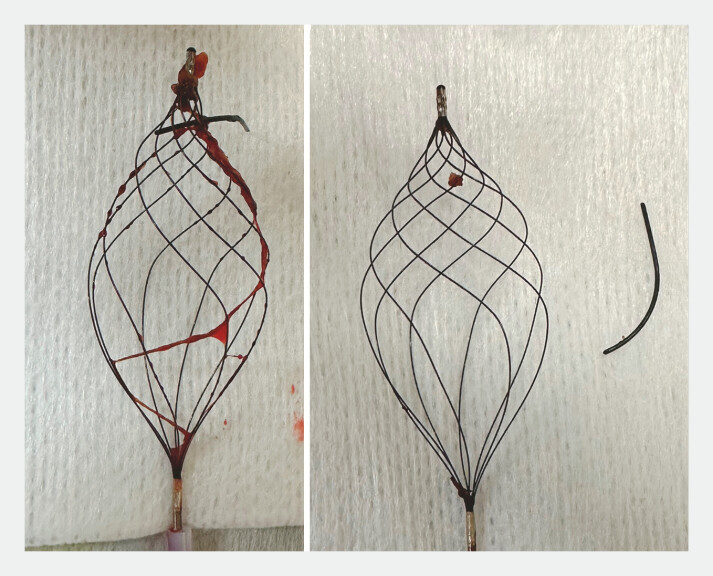
The sheared guidewire remnant and basket catheter.

There have been few reports in the literature concerning techniques for retrieving residual guidewires, mostly using biopsy forceps during endoscopic ultrasound or ERCP 2 3 . To our knowledge, no studies have yet described remnant guidewire retrieval in the gallbladder duct using a basket catheter. This new type of basket catheter has a unique shape and rotational mechanism that may be useful not only for removing bile duct stones but also for retrieving foreign bodies in narrow areas such as the gallbladder duct ( Video 1 ).

Video showing the retrieval of a sheared guidewire remnant in the gallbladder duct using the novel RASEN 2 basket catheter.Video 1

Endoscopy_UCTN_Code_TTT_1AR_2AG

## Notes

Endoscopy E-Videos https://eref.thieme.de/e-videos
*E-Videos* is an open access online section of the journal *Endoscopy* , reporting on interesting cases and new techniques in gastroenterological endoscopy. All papers include a high-quality video and are published with a Creative Commons CC-BY license. Endoscopy E-Videos qualify for HINARI discounts and waivers and eligibility is automatically checked during the submission process. We grant 100% waivers to articles whose corresponding authors are based in Group A countries and 50% waivers to those who are based in Group B countries as classified by Research4Life (see: https://www.research4life.org/access/eligibility/ ). This section has its own submission website at https://mc.manuscriptcentral.com/e-videos .
